# Co-Delivery of Glucose Oxidase and Iron-Doped ZIF-8 as a pH-Responsive Ferroptosis and Starvation Agent for Triple-Negative Breast Cancer Therapy

**DOI:** 10.3390/nano16090533

**Published:** 2026-04-28

**Authors:** Zhibin Lin, Yuanxin Zhao, Lin Tang, Jianhua He

**Affiliations:** The Institute for Advanced Studies, Wuhan University, Wuhan 430072, China; 2019302020065@whu.edu.cn (Z.L.); tanglin@whu.edu.cn (L.T.)

**Keywords:** ZIF-8, glucose oxidase, ferroptosis, starvation therapy, triple-negative breast cancer

## Abstract

Currently, single-modal tumor therapy has significant limitations, while multi-modal combination therapy can overcome this bottleneck and open up new pathways for enhancing the efficacy of tumor therapy. However, it is still difficult to design a functionalized nanocarrier that can simultaneously mediate multiple therapeutic approaches. To tackle this challenge, we developed a multifunctional nano-codelivery system with glucose oxidase (GOx) loaded inside iron-doped zeolitic imidazolate framework-8 (Fe/ZIF-8), abbreviated as GFZ. This system effectively integrates the synergy and complementarity between ferroptosis therapy and starvation therapy (STT). Herein, GFZ innovatively combines the pH sensitivity of the ZIF-8 skeleton with the EPR effect of nanoparticles to achieve on-demand triggered release, significantly improving the accuracy of tumor targeting. Furthermore, GOx-mediated STT effectively alleviates the insufficiency of endogenous H_2_O_2_ during the ferroptosis process, thereby enhancing and synergizing with ferroptosis therapy. Experiments demonstrated both in vitro and in vivo that GFZ activates antitumor cascade reactions, inhibits tumor recurrence and metastasis, and exhibits excellent biocompatibility. Consequently, given its remarkable potential, GFZ is poised to emerge as a new mode of nano-delivery platform.

## 1. Introduction

Cancer has been one of the most harmful diseases to humanity for decades, with no complete solution yet available [1]. Numerous scientific teams are researching cancer treatments. Chemotherapy is currently one of the most common clinical treatment methods and has remained the cornerstone of pharmacological treatment for malignant tumors since its emergence in the mid-20th century. Its core mechanism lies in using cytotoxic drugs to interfere with or block key processes in the tumor cell proliferation cycle, inducing cell apoptosis or death and thereby inhibiting and eliminating malignant tumors [2,3].

However, the targets of chemotherapy drugs are not highly specific. While killing rapidly proliferating tumor cells, they inevitably cause varying degrees of damage to normal tissues and cells with active proliferation in the body, leading to a series of treatment-related toxic reactions [4,5]. These side effects not only directly affect patients’ treatment tolerance and quality of life, but also result in treatment interruption, dose adjustment, and secondary complications, becoming key factors limiting the maximization of chemotherapy efficacy and its widespread clinical application. To address the drawbacks of chemotherapy and better treat malignant tumors, novel tumor therapy methods have emerged [6,7].

Ferroptosis [8] is characterized by iron-dependent accumulation of lethal lipid peroxides (LPO) and is distinct from other known death mechanisms such as apoptosis [9], necrosis [10], and autophagy [11]. The concept was first explicitly proposed in 2012 by the laboratory of Brent R. Stockwell, marking a significant breakthrough in the study of cell death [12]. The core mechanism of ferroptosis lies in the iron ion-induced Fenton reaction, which produces a substantial amount of hydroxyl radicals, thereby initiating lipid peroxidation. This process involves the inactivation of the glutathione peroxidase 4 (GPX4) antioxidant defense system, metabolic abnormalities in the intracellular free iron pool, and uncontrolled peroxidation of membrane phospholipids containing unsaturated fatty acids, ultimately leading to the loss of cell membrane system integrity. Some studies have found that certain malignant cells with high metastatic potential or treatment-resistant characteristics exhibit unique sensitivity to ferroptosis [13,14]. This provides a highly promising new target for overcoming the resistance bottlenecks of traditional treatments.

Starvation therapy is also an emerging treatment approach in recent years [15,16]. Its core principle lies in achieving specific tumor inhibition by disrupting the tumor’s nutrient supply system and targeting its metabolic dependencies [17]. In 1971, Harvard University Professor Judah Folkman first proposed the anti-angiogenesis theory, laying the theoretical foundation for starvation therapy. Traditional starvation therapy primarily relies on anti-angiogenic drugs to inhibit the formation of new blood vessels within tumors [18]. Due to the Warburg effect, a phenomenon commonly observed in cancer cells, tumor cells preferentially consume large amounts of glucose through the glycolysis pathway even under oxygen-sufficient conditions [19]. Against this backdrop, glucose oxidase (GOx), as a natural biocatalyst, has entered the research landscape of tumor therapy. GOx efficiently catalyzes the reaction between β-D-glucose and oxygen to produce gluconic acid and hydrogen peroxide [20,21]. This unique property positions it as an ideal tool for realizing starvation therapy.

However, single novel therapeutic approaches often yield limited efficacy due to the complexity of the tumor microenvironment (TME) and adaptive compensatory mechanisms [22]. Current research increasingly emphasizes combination strategies integrating multiple novel treatment modalities [23]. For instance, the hyperthermia induced by photothermal therapy (PTT) can enhance the permeability of tumor cell membranes, thereby facilitating the uptake of chemotherapeutic drugs [24]. Radiotherapy primarily targets the G2/M phase of the cell cycle, while chemotherapeutic agents can act on tubulin to arrest cells in the G2/M phase, thereby sensitizing them to radiotherapy [25]. To achieve the combination of multiple therapeutic modalities, a mediator is required, and nanomaterials, leveraging their unique size effects, high specific surface area, and designable functionalities, offer revolutionary tools for constructing intelligent drug delivery systems and multifunctional therapeutic platforms [26,27].

Metal–organic frameworks (MOFs) represent a class of crystalline, porous coordination polymers formed from metal nodes and organic struts. Their extraordinarily large surface area, controllable pore dimensions, straightforward post-synthetic modification, and outstanding biocomposition position them as highly attractive candidates for drug delivery applications [28,29]. And MOFs can not only serve as delivery carriers but also participate in therapy as functional components [30,31]. Yang et al. successfully fabricated CQ-loaded NH_2_-MIL-88B(Fe) nanoparticles with a size of approximately 200 nm. This platform triggers ferroptosis through the introduction of Fe ions, while simultaneously releasing CQ to inhibit the autophagic process, effectively elevating intracellular hydroxyl radical (·OH) levels and achieving enhanced antitumor efficacy [32]. Zhao et al. successfully constructed an Mn-doped MOF nanoplatform loaded with the antitumor drug FOE (DUCNP@Mn-MOF/FOE). By combining the pH-responsive and peroxidase-mimicking features of Mn-MOF with the distinctive optical characteristics of DUCNPs, this platform achieves a synergistic interplay between chemodynamic and chemotherapeutic actions. It exhibited excellent tumor targeting capability and successfully circumvented the inherent drawbacks of FOE, including its unsatisfactory physicochemical properties and insufficient in vivo efficacy [33].

Therefore, this study aims to construct a multifunctional cascade-responsive nanotheranostic platform. The platform uses pH-responsive degradable zeolitic imidazolate framework-8 (ZIF-8) as the core carrier, incorporates iron salts during synthesis for iron doping, and simultaneously encapsulates hydrophilic GOx. This design is expected to achieve the following functions: (1) utilize the porous structure of ZIFs to achieve higher drug loading efficiency and aqueous dispersion stability; (2) leverage the acid-responsive dissociation and “proton sponge” effect of ZIFs to achieve intelligent drug release and efficient cytoplasmic delivery in tumor cell lysosomes, promoting nuclear accumulation of the drug to exert telomerase inhibition; (3) the iron ions released from the degradation of the ZIFs framework can trigger Fenton reactions in tumor cells, inducing lipid peroxidation and ferroptosis; (4) the GOx released from the degradation of the ZIFs framework can deplete glucose within tumor cells, triggering starvation therapy. Through this dual therapeutic approach of “carrier degradation-induced ferroptosis” and “drug release-mediated glucose depletion”, a multi-pathway precise attack on tumor cells can be achieved. This study not only provides a new strategy for overcoming the clinical application bottlenecks of glucose oxidase but also offers new insights for developing MOF-based synergistic therapeutic nanoplatforms.

## 2. Materials and Methods

### 2.1. Synthesis of ZIF-8 NPs

The ZIF-8 NPs were prepared by the solvothermal method. First, 32 mmol 2-methylimidazole (2-MI) was dissolved in 25 mL DI, and 1 mmol Zn(NO_3_)_2_·6H_2_O was dissolved in 10 mL DI. The Zn(NO_3_)_2_ solution was gradually introduced drop by drop into the 2-MI solution, followed by stirring at 500 rpm for 30 min at ambient temperature. After centrifugation, the resulting precipitate was washed three times with DI water, then transferred to an oven (WGLL-45BE, Huanghua Faithful Instrument Co., Ltd., Huanghua, China) and dried at 45 °C overnight.

### 2.2. Synthesis of Fe/ZIF-8 NPs

For this step, 32 mmol 2-MI was dissolved in 25 mL DI; then, 0.9 mmol Zn(NO_3_)_2_·6H_2_O and 0.1 mmol FeSO_4_·7H_2_O were dissolved in 10 mL DI. The latter solution was gradually introduced drop by drop into the 2-MI solution, followed by stirring at 500 rpm for 30 min at ambient temperature. After centrifugation, the resulting precipitate was washed three times with DI water, then transferred to an oven and dried at 45 °C overnight.

### 2.3. Synthesis of GOx@Fe/ZIF-8 NPs

For this step, 32 mmol 2-MI and 30 mg GOx were dissolved in 25 mL DI; then, 0.9 mmol of Zn(NO_3_)_2_·6H_2_O and 0.1 mmol FeSO_4_·7H_2_O were dissolved in 10 mL DI. The latter solution was gradually introduced drop by drop into the 2-MI solution, followed by stirring at 500 rpm for 30 min at ambient temperature. After centrifugation, the resulting precipitate was washed three times with DI water, then transferred to an oven and dried at 45 °C overnight.

### 2.4. Study of Drug Release Behavior

To study how pH affects the release behavior of GOx, GOx@Fe/ZIF-8 was suspended in PBS (pH 5.4) and then maintained at 37 °C under mild agitation at 200 rpm. At predetermined time intervals, samples were collected, centrifuged, and the supernatant was harvested. After each sampling, the same volume of fresh PBS was added back to the system. The collected supernatant was extracted with deionized water, and its absorbance was measured over a wavelength range of 200–800 nm using a UV spectrophotometer (UV-2550, Shimadzu Corporation, Kyoto, Japan). The concentration of released GOx was determined using a standard curve.

### 2.5. Study of ROS Generation Behavior by NPs

To investigate the intrinsic ability of the materials to generate hydroxyl radicals, a mixed solution of TMB and hydrogen peroxide was prepared using a buffer at pH 4. ZIF-8, Fe/ZIF-8, GOx@ZIF-8, and GOx@Fe/ZIF-8 at specific concentrations were dispersed in the TMB mixed solution and maintained at 37 °C for a duration of 30 min. After centrifugation, the supernatant was collected, and the absorbance of the sample was scanned between 200 and 900 nm with a UV spectrophotometer.

### 2.6. Cytotoxicity Assay

Cells were cultured in RPMI-1640 medium supplemented with 10% fetal bovine serum (FBS) and 1% penicillin-streptomycin (P/S). Each well of the 96-well plate was dispensed with 4T1 cells at a seeding density of 5000 cells. After 24 h, the cells became fully adherent. The original medium was then replaced with fresh medium containing various concentrations (0, 12.5, 25, 50, 100, and 200 μg/mL) of ZIF-8, Fe/ZIF-8, GOx@ZIF-8, and GOx@Fe/ZIF-8, and the cells were incubated for an additional 24 h. A microplate reader (SpectraMax iD5, Molecular Devices LLC., San Jose, CA, USA) was employed to measure the absorbance of each well at 570 nm according to the MTT protocol, from which the relative viability of the cells was derived.

### 2.7. Cell Apoptosis Assay

Each well of the 6-well plate was dispensed with 4T1 cells at a seeding density of 300,000 cells. After 24 h, the cells became fully adherent. The original medium was then replaced with fresh medium containing specific concentrations (100 μg/mL) of ZIF-8, Fe/ZIF-8, GOx@ZIF-8, and GOx@Fe/ZIF-8, and the cells were incubated for an additional 24 h. An Annexin V-FITC/PI apoptosis detection kit was employed to stain the cells according to the manufacturer’s protocol, from which the apoptosis rate of each group was derived.

### 2.8. Cellular ROS Assay

Each well of the 6-well plate was dispensed with 4T1 cells at a seeding density of 150,000 cells. After 24 h, the cells became fully adherent. The original medium was then replaced with fresh medium containing specific concentrations (100 μg/mL) of ZIF-8, Fe/ZIF-8, GOx@ZIF-8, and GOx@Fe/ZIF-8, and the cells were incubated for an additional 24 h. The cells were rinsed with serum-free RPMI-1640 medium, followed by the addition of 1 mL of DCFH-DA probe (10 nmol/mL) to each well. All 6-well plates were placed in a thermostatic incubator and further incubated for 30 min in the dark. CLSM (CKX41, Olympus Corporation, Tokyo, Japan) was used to visualize the intracellular ROS levels at an excitation wavelength of 488 nm. while flow cytometry (NovoCyte 2070R, Agilent Technologies Inc., Santa Clara, CA, USA) was applied to quantify them.

### 2.9. Intracellular GSH Assay

Each well of the 6-well plate was dispensed with 4T1 cells at a seeding density of 300,000 cells. After 24 h, the cells became fully adherent. The original medium was then replaced with fresh medium containing specific concentrations (100 μg/mL) of ZIF-8, Fe/ZIF-8, GOx@ZIF-8, and GOx@Fe/ZIF-8, and the cells were incubated for an additional 24 h. After trypsinization and collection of cells, the cell lysates were transferred to a 96-well plate. A microplate reader was employed to measure the absorbance of each well at 412 nm using GSH and GSSG assay kits, from which the intracellular GSH content was derived.

### 2.10. Intracellular Fe^2+^ Assay

Each well of the 6-well plate was dispensed with 4T1 cells at a seeding density of 150,000 cells. After 24 h, the cells became fully adherent. The original medium was then replaced with fresh medium containing specific concentrations (100 μg/mL) of ZIF-8, Fe/ZIF-8, GOx@ZIF-8, and GOx@Fe/ZIF-8, and the cells were incubated for an additional 24 h. After trypsinization and collection of cells, the cell lysates were transferred to a 96-well plate. A microplate reader was employed to measure the fluorescence intensity of each well at 593 nm using a Ferro Orange assay kit, from which the intracellular Fe^2+^ content was derived.

### 2.11. Intracellular MDA Assay

Each 10 cm culture dish was dispensed with 4T1 cells at a seeding density of 1,500,000 cells. After 24 h, the cells became fully adherent. The original medium was then replaced with fresh medium containing specific concentrations (100 μg/mL) of ZIF-8, Fe/ZIF-8, GOx@ZIF-8, and GOx@Fe/ZIF-8, and the cells were incubated for an additional 24 h. After trypsinization and collection of cells, the cell lysates were mixed with an equal volume of thiobarbituric acid (TBA) working solution. The mixture was then heated in a boiling water bath for 45 min, cooled to room temperature, and transferred to a 96-well plate. A microplate reader was employed to measure the absorbance of each well at 532 nm, from which the intracellular MDA content was derived.

### 2.12. Animals and Tumor Models

All animal experiments were conducted in accordance with the *Guide for the Care and Use of Laboratory Animals (8th edition),* and ethical approval was obtained prior to the experiments. Female Balb/c mice with similar growth status, aged 4–6 weeks and weighing approximately 18–20 g, were selected for the experiments. Murine 4T1 triple-negative breast cancer cells were suspended in PBS at a concentration of 5 × 10^6^ cells/mL. A subcutaneous tumor-bearing model was successfully established by subcutaneously injecting 200 μL of 4T1 cell suspension into the right hind leg of each mouse.

### 2.13. Hemolysis Assay

This experiment was conducted following established protocols. Briefly, 5 mL of blood samples were collected from Balb/c mice. Fibrinogen was eliminated through vigorous stirring. The resulting product was then washed three times with PBS at a volume of approximately 10 times that of the sample. Subsequently, a 2% erythrocyte suspension was prepared in PBS. Then, 0.5 mL of the erythrocyte suspension was mixed with varying concentrations of ZIF-8 and GOx@Fe/ZIF-8 in test tubes, achieving final material concentrations of 25, 50, 100, 150, 200, 300, and 400 μg/mL. Positive controls and negative controls were included. Incubation of all tubes was carried out at 37 °C for 3 h.

### 2.14. In Vivo Tumor Inhibition Experiment

After successfully establishing the 4T1 triple-negative breast cancer model and reaching tumor volumes of approximately 100 mm^3^, mice were randomly assigned to four groups, with five mice in each group. The mice in each group were intravenously injected with PBS, ZIF-8, GOx@Fe/ZIF-8, or GOx every three days over a period of 16 days, corresponding to a total of five injections. Body weight, tumor dimensions, and the overall health status of the mice were recorded every 3 days. Tumor volume was determined according to the equation V = (A × B^2^)/2, where A is the longest tumor axis and B is the short axis perpendicular to A. Upon completion of the experiment, all mice were euthanized under deep anesthesia following intraperitoneal injection of pentobarbital sodium (150 mg/kg). The tumor tissues and major organs (spleen, lungs, heart, liver, and kidneys) were collected. After weighing and photographing, the specimens were fixed in 4% neutral formaldehyde for hematoxylin and eosin (H&E) staining. Before staining, all samples were embedded in tissue-freezing medium and cryopreserved at −80 °C.

## 3. Results and Discussion

### 3.1. NP Synthesis and Characterization

**Figure 1:**
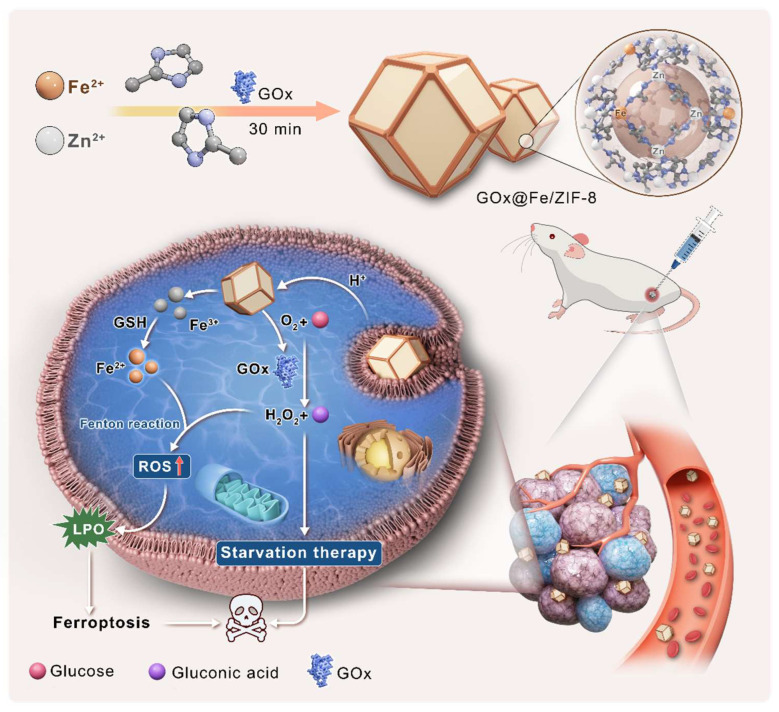
Schematic diagram illustrating the synthesis of GOx@Fe/ZIF-8 and the mechanism by which it enhances ferroptosis.

The synthesis process and therapeutic mechanism of GOx@Fe/ZIF-8 (GFZ) are illustrated in Figure 1. We adopted a one-pot method to directly synthesize GFZ and evaluated its therapeutic mechanism.

**Figure 2:**
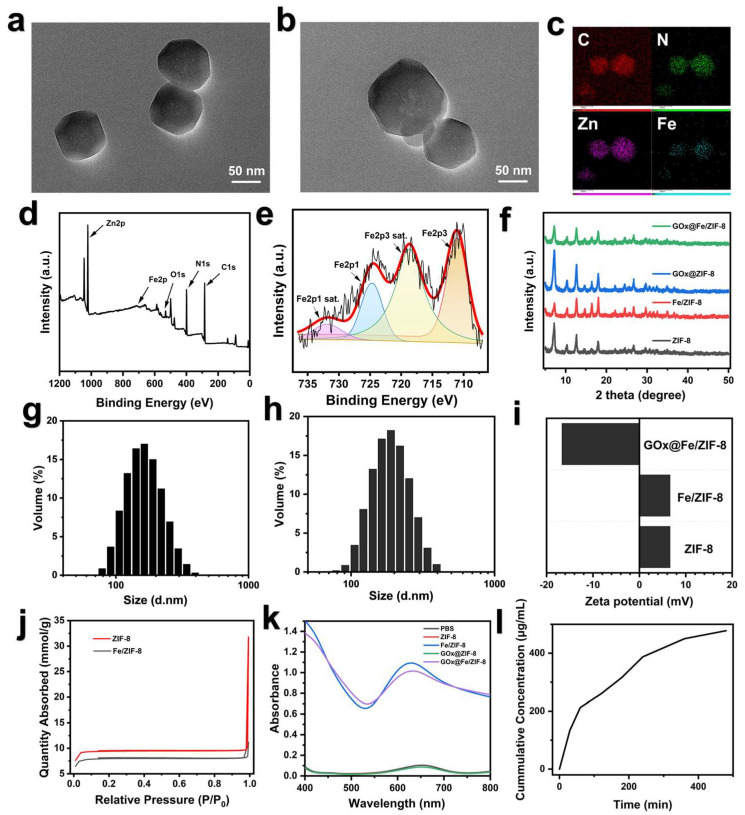
(**a**) TEM image of ZIF-8, (**b**) TEM image, (**c**) EDS elemental mapping of Fe/ZIF-8, (**d**) XPS survey spectrum of Fe/ZIF-8, (**e**) Fe 2p XPS spectrum of Fe/ZIF-8, (**f**) X-ray diffraction (XRD) patterns of ZIF-8, Fe/ZIF-8, GOx@ZIF-8, and GOx@Fe/ZIF-8, (**g**) particle size distribution of Fe/ZIF-8, (**h**) particle size distribution of GOx@Fe/ZIF-8, (**i**) zeta potentials of ZIF-8, Fe/ZIF-8, and GOx@Fe/ZIF-8, (**j**) nitrogen adsorption–desorption isotherms of ZIF-8 and Fe/ZIF-8, (**k**) UV-Vis absorption spectra of different materials dispersed in water with H_2_O_2_ and TMB, (**l**) cumulative release concentration of GOx from GOx@Fe/ZIF-8 at pH 5.4.

Transmission electron microscopy (TEM) results showed that both ZIF-8 NPs and iron-doped ZIF-8 NPs (Fe/ZIF-8) displayed excellent dispersion, consistent particle dimensions, and well-defined surface morphology (Figure 2a,b). Meanwhile, the elemental mapping results in Figure 2c revealed the presence of iron in Fe/ZIF-8 (FZ) and demonstrated that Fe, Zn, C, and N were homogeneously dispersed across the entire material. This phenomenon confirmed the successful doping of iron ions. Figure 2d presents the XPS full spectrum of FZ. A distinct absorption peak of Fe can be clearly observed in the energy range of 700–800 eV. Additionally, the XPS spectrum in the Fe 2p region exhibits the typical dual-peak structure of iron species (Fe 2p3/2 and Fe 2p1/2), confirming the presence of iron in the material (Figure 2e). The XRD results confirm the successful synthesis of the ZIF-8 material, and the loading of GOx has no impact on its crystalline structure. The incorporation of Fe ions reduces the intensity of the (1, 0, 0) peak of ZIF-8 while inducing a slight rightward shift, which is attributed to the difference in ionic radii between Fe and Zn ions (Figure 2f).

The particle size distribution results shown in Figure 2g,h indicate that the peak particle sizes of FZ and GOx@Fe/ZIF-8 (GFZ) are 171.1 nm and 196.6 nm, respectively. Compared with ZIF-8, FZ exhibited a smaller particle size, which may be attributed to lattice instability caused by Fe ion doping that inhibits crystal growth. The increased particle size of GFZ confirms the successful loading of GOx. Additionally, the zeta potential data in Figure 2g show that both ZIF-8 and FZ exhibit positive zeta potentials of approximately +6.61 mV, while GFZ displays a zeta potential of about −16.67 mV, further demonstrating the successful loading of GOx. As evidenced by the N_2_ adsorption–desorption isotherms, both ZIF-8 and FZ exhibit typical Type I isotherm characteristics, indicating the presence of a microporous structure in the materials (Figure 2j). The pore size of GOx@Fe/ZIF-8 is 1.4912 nm, while that of ZIF-8 is 1.4775 nm. The specific surface area of GOx@Fe/ZIF-8 (752.3657 m^2^/g) is slightly lower than that of ZIF-8 (900.8884 m^2^/g). The above results verify that GOx@Fe/ZIF-8 was successfully fabricated.

### 3.2. Evaluation of ROS Generation Capacity and pH Responsiveness of NPs

To explore the potential of GOx@Fe/ZIF-8 for antitumor applications by enhancing ferroptosis, this study first investigated the effect of pH on the release behavior of GOx from the composite. As shown in Figure 2i, the release of GOx exhibited typical time- and pH-dependent characteristics. In an acidic environment (pH 5.4) simulating tumor cell lysosomes, GFZ demonstrated rapid and efficient responsive release: at just 30 min, the release concentration of GOx reached 134 μg/mL. Over time, the cumulative release continued to increase, reaching approximately 450 μg/mL and 478 μg/mL at 6 h and 8 h, respectively, achieving a nearly 100% cumulative release rate. This result strongly demonstrates that the GFZ nanoplatform possesses excellent drug-controlled-release capability in the acidic tumor microenvironment, ensuring effective enrichment of GOx at the target site.

Furthermore, the •OH generation capacity of four materials was systematically evaluated using the TMB oxidation assay. As depicted in Figure 2k, the ZIF-8 alone and GOx-loaded GZ groups exhibited only weak absorption peaks at 652 nm, indicating their limited Fenton reaction capability. In contrast, both the iron-doped FZ and GFZ groups displayed significant absorption peaks at the same wavelength. This key difference clearly indicates that the successful doping of Fe elements is a prerequisite for endowing the platform with •OH generation capability. More importantly, in conjunction with the aforementioned GOx release behavior, GFZ can not only respond to the acidic environment by releasing GOx to consume glucose, but its doped iron ions can also efficiently catalyze the Fenton reaction to generate highly toxic •OH.

### 3.3. In Vitro Antitumor Effect and Mediation of Cellular Ferroptosis

**Figure 3:**
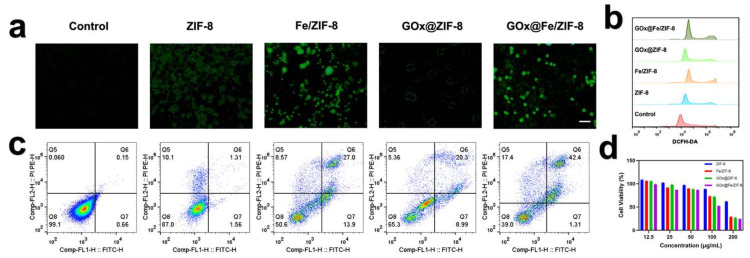
(**a**) Representative images (scale bar: 50 μm), (**b**) flow cytometric analysis of ROS in 4T1 cells after different treatments, (**c**) flow cytometry results of cell viability in 4T1 cells after different treatments, (**d**) viability of 4T1 cells after 24 h of treatment with various concentrations and different conditions.

The in vitro biocompatibility and therapeutic efficacy of the materials were evaluated via MTT assays. As shown in Figure 3d, for 4T1 murine breast cancer cells, FZ, GZ, and GFZ all exhibited concentration-dependent cytotoxicity when the material concentration increased from 12.5 μg/mL to 100 μg/mL, with cell viability decreasing progressively. Notably, GOx synergistically enhanced the therapeutic effect of FZ, reducing 4T1 cell viability to 60% under optimal conditions. Concurrently, Figure 3c presents the quantitative analysis of the live/dead cell ratio determined by flow cytometry, which was performed simultaneously. At a material concentration of 100 μg/mL, nearly no late apoptotic cells were observed in either the control group or the ZIF-8 group. In contrast, the FZ and GZ groups show death rates of 27.0% and 20.3%, respectively. Notably, the GFZ group demonstrated the highest proportion of dead cells at 42.4%, significantly exceeding those of the FZ and GZ groups. This combined starvation-ferroptosis therapy demonstrated significantly superior efficacy compared to individual treatment modalities.

To assess the ROS-generating capacity of various materials, 4T1 cells were incubated with the fluorogenic dye DCFH-DA. As shown in Figure 3a, no obvious green fluorescence from DCFH-DA was observed in the control group, while only weak green fluorescence appeared in the ZIF-8 and GZ groups. In contrast, pronounced green fluorescence signals were evident in both the FZ and GFZ groups, indicating that these composite materials could effectively generate ROS in 4T1 cells. Meanwhile, flow cytometry combined with the DCFH-DA probe was employed to quantitatively assess ROS generation in cells treated with different materials. As shown in Figure 3b, the results were highly consistent with the observations from confocal microscopy. Flow cytometric analysis further demonstrated that the fluorescence intensity of the GFZ group exceeded that of the FZ group, indicating that the synergistic effect of GOx confers enhanced ROS-generating capacity to GFZ.

**Figure 4:**
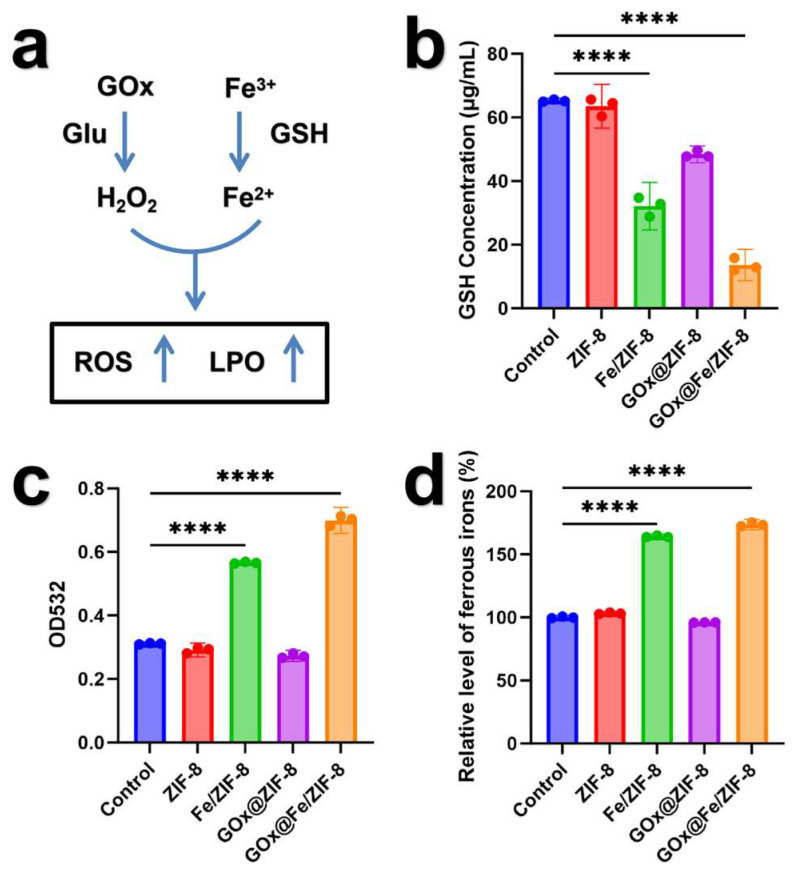
(**a**) Schematic diagram illustrating the molecular mechanism of synergistic therapy involving starvation treatment and ferroptosis, (**b**) GSH levels, (**c**) MDA levels, (**d**) ferrous levels in 4T1 cells after different treatments. n = 3. **** *p* < 0.0001.

Lipid peroxidation (LPO), glutathione (GSH), and ferrous ions are critical markers of ferroptosis. Figure 4a illustrates the molecular mechanism of the synergistic therapy involving starvation and ferroptosis induced by GFZ nanomaterials. Figure 4b displays the changes in intracellular GSH levels after treatment with different groups. The GFZ group exhibits the lowest GSH levels, while the FZ group also significantly reduces intracellular GSH content. This indicates that the iron ions in the materials successfully enter the cells and react effectively with GSH.

Malondialdehyde (MDA) is a product of lipid peroxidation (LPO) generated during ferroptosis. Figure 4c shows the intracellular MDA levels after treatment with different materials. The MDA content in the GFZ group is 2.5 times that of the control group, while the FZ group exhibits twice the MDA level of the control. In contrast, the groups without iron ions (GZ and ZIF-8 groups) show MDA levels comparable to the control. The composite materials exhibited a remarkable ability to induce MDA production, as evidenced by these results.

Figure 4d likewise demonstrates that the ferrous ion levels in the GZ and ZIF-8 groups are comparable to those in the control group. In contrast, the intracellular ferrous ion content in the GFZ and FZ groups significantly exceeded normal levels, with the GFZ group showing a 1.8-fold increase and the FZ group showing a 1.7-fold increase compared to the control. These results indicate that the pH-responsive composite materials successfully entered tumor cells and disintegrated under TME.

These results show that the composite material is effectively endocytosed into the cells and successfully hydrolyzed within the weakly acidic tumor microenvironment, releasing Fe ions and GOx from the ZIF framework. Through the combined mechanisms of starvation therapy and ferroptosis, GOx@Fe/ZIF-8 exhibits significant antitumor efficacy at the cellular level.

### 3.4. Tumor Suppression Evaluation In Vivo

**Figure 5:**
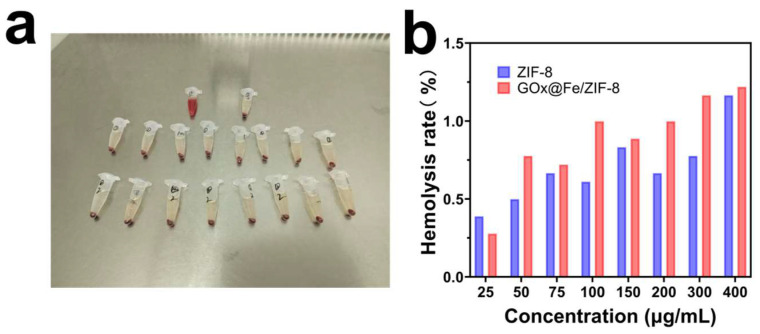
In vivo hemolysis evaluation of ZIF-8 and GOx@Fe/ZIF-8. (**a**) Photographs of blood tubes after centrifugation for each concentration group; (**b**) hemolysis rate bar graph after treatment with varying concentrations of materials.

Given that we observed enhanced cytotoxicity of GFZ NPs in vitro, we further evaluated their tumor-suppressing efficacy in vivo. First, we assessed the biosafety and biocompatibility of GFZ by performing a hemolysis assay. As shown in Figure 5, when mouse red blood cells were extracted and incubated with varying concentrations of GFZ, the results showed that even at a material concentration of 400 μg/mL, the hemolysis rate remained below 5%. This demonstrated that GFZ NPs are biologically safe.

**Figure 6:**
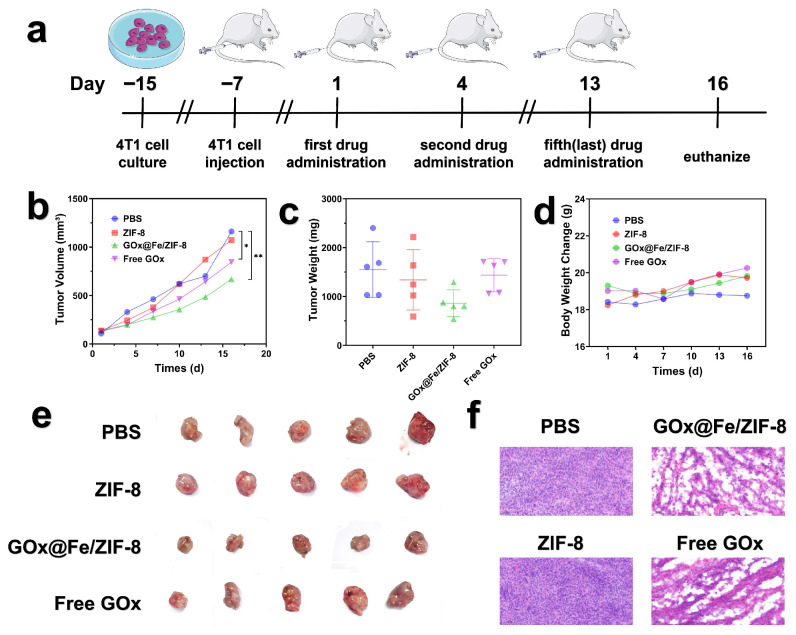
(**a**) Schematic illustration of the animal experimental treatment, (**b**) changes in tumor volume over the course of treatment (two-way ANOVA followed by Tukey’s multiple comparison test), (**c**) the weights of excised tumors after treatment (one-way ANOVA followed by Tukey’s multiple comparison test), (**d**) the body weight fluctuations of mice over the course of treatment, (**e**) photographs of excised tumors, (**f**) H&E staining images of tumor tissues after different treatments. n = 5. * *p* < 0.05, ** *p* < 0.01.

Subsequently, we evaluated the tumor-suppressing capability of GFZ and constructed an orthotopic mouse model of 4T1 tumor cells, as illustrated in Figure 6a. An orthotopic xenograft tumor model is established by directly injecting tumor fluid into the subcutaneous region of the right leg using a fine needle. Tumor-bearing mice were randomized into four groups: control, ZIF-8, GFZ, and free GOx. On day 16 after injection, the tumor volume in the ZIF-8 group is comparable to that of the control group, while the free GOx and GFZ groups show reductions by factors of 0.43 and 0.70, respectively, indicating potent tumor suppression in the GFZ group (Figure 6b).

Figure 6e shows the excised tumor masses from all mice after completion of the treatment regimen. Compared with the other groups, mice in the GFZ treatment group were observed to have substantially reduced tumor sizes.

Moreover, the harvested tumor specimens were subjected to weight measurement. The tumor weights in the control, ZIF-8, GFZ, and free GOx groups are 1551 mg, 1427 mg, 862 mg, and 1435 mg, respectively (Figure 6c). However, the difference in tumor-suppressive effects between ZIF-8 and free GOx was not particularly significant, which may be attributed to the poor targeting ability of free GOx. Notably, throughout the treatment period, none of the four groups exhibited significant body weight fluctuations, further supporting the biosafety of the treatment (Figure 6d).

**Figure 7:**
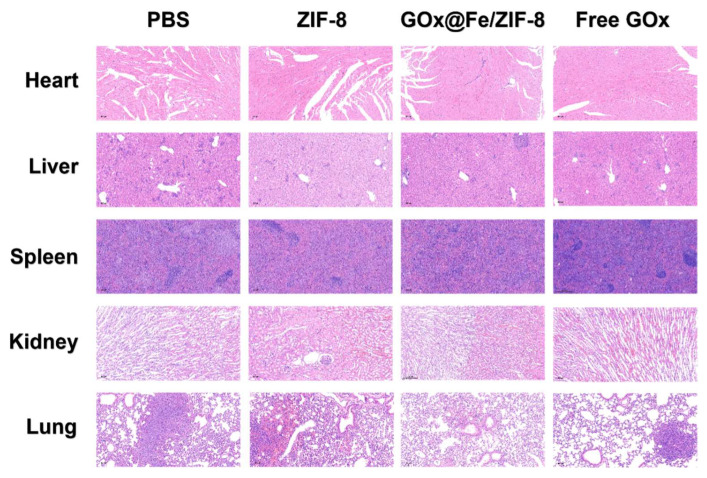
H&E-stained sections of major organs from healthy mice following various treatments. Acquisition magnifications: 10× for heart, 10× for liver, 5× for spleen, 10× for kidney, 10× for lung.

Tumor tissue sections from mice are subjected to staining and imaging analysis. Hematoxylin-eosin (H&E) staining revealed densely packed tumor cells in the control and ZIF-8 groups. In contrast, small foci of apoptotic and necrotic cells were detected in the free GOx group, whereas extensive areas of apoptosis and necrosis were observed in the GFZ group (Figure 6f). Meanwhile, H&E staining revealed no significant histopathological alterations in the major organs (heart, liver, spleen, lung, and kidney) of mice across all treatment groups (Figure 7). Specifically, cardiac muscle fibers were regularly arranged with no evidence of cardiomyocyte necrosis or inflammatory infiltration. The liver exhibited intact lobular architecture with radially oriented hepatocyte cords, and no hepatocyte edema, steatosis, or necrotic foci were observed. The spleen displayed a clear demarcation between the red and white pulp with preserved splenic corpuscles, and no lymphocytic depletion or fibrosis was noted. Lung tissue showed intact alveolar structures without apparent septal thickening, inflammatory exudate, or pulmonary edema. The kidney presented well-defined glomeruli and normal renal tubular epithelial morphology, with no glomerulosclerosis or tubular necrosis. Collectively, these results demonstrate that the material does not induce detectable toxicity in major organs at the therapeutic dose, indicating favorable in vivo biosafety.

## 4. Conclusions

Herein, we developed a GOx-loaded iron-doped ZIF-8 nanodrug and proposed a combined therapeutic strategy integrating ferroptosis and starvation therapy for the treatment of triple-negative breast cancer. In vitro experimental results demonstrated that this nanodrug exhibits excellent cancer cell-killing efficacy. More importantly, an in-depth investigation was conducted into the mechanism underlying its induction of tumor cell death. This nanodrug delivery system is capable of depleting intracellular glutathione (GSH), promoting the generation of lipid peroxidation (LPO), and simultaneously achieving sustained iron ion supply. In vivo experiments performed on a xenograft mouse model bearing 4T1 cells verified that this nanodrug effectively suppresses tumor growth while exhibiting a favorable safety profile.

Although there are still limitations, such as low iron ion doping efficiency, insufficient glucose oxidase loading capacity, and nanoparticle aggregation at high concentrations, this study provides a preliminary proof of concept for the synergistic therapeutic strategy combining ferroptosis and starvation therapy. Future research will focus on optimizing the synthesis process to improve iron ion doping efficiency, exploring surface modification strategies to enhance the dispersion stability of nanoparticles under physiological conditions, and further validating their targeted accumulation in tumor tissues using fluorescence imaging and other approaches. We believe that this therapeutic strategy holds potential for significant therapeutic benefits and warrants further in-depth exploration.

## Data Availability

The data and materials generated and analyzed during this paper can be obtained from the corresponding authors upon reasonable request.
